# Case Report: *ASXL1*, *RUNX1*, and *IDH1* mutation in tyrosine kinase-independent resistant chronic myeloid leukemia progressing to chronic myelomonocytic leukemia-like accelerated phase

**DOI:** 10.3389/fonc.2023.1217153

**Published:** 2023-09-07

**Authors:** Emmanuella Oyogoa, Lukas Streich, Philipp W. Raess, Theodore Braun

**Affiliations:** ^1^ Department of Medicine, Oregon Health & Science University, Portland, OR, United States; ^2^ Department of Pathology, Oregon Health & Science University, Portland, OR, United States; ^3^ Division of Hematology & Medical Oncology, Oregon Health & Science University, Portland, OR, United States

**Keywords:** CML, tyrosine kinase inhibitors, ASXL1, RUNX1, CMML-AP, systemic inflammatory and autoimmune diseases, tyrosine kinase independent resistance

## Abstract

Although the majority of patients with chronic myeloid leukemia (CML) enjoy an excellent prognosis tyrosine kinase inhibitor (TKI) therapy, resistance remains a significant clinical problem. Resistance can arise from mutations in the kinase domain of ABL preventing drug binding, or due to ill-defined kinase-independent mechanisms. In this case report, we describe the case of a 27-year-old woman with a long-standing history of chronic phase (CP) CML who developed kinase-independent resistance with mutations in *ASXL1* and *RUNX1*. As a consequence of uncontrolled disease, she progressed to a chronic myelomonocytic leukemia-like (CMML) accelerated phase (AP) disease with the acquisition of a mutation in *IDH1*. This disease progression was associated with the development of an inflammatory serositis, a phenomenon that has been described in CMML but not in AP-CML. This case presents key features of kinase-independent resistance with insight into potential mechanisms, highlights management challenges, and describes a novel systemic inflammatory response that occurred in this patient upon disease progression.

## Introduction

Chronic myeloid leukemia (CML) is a hematologic malignancy caused by the translocation t(9;22)(q34;q11) that fuses the break point cluster region gene (*BCR*) on chromosome 22 to the Abelson Murine Leukemia-(*ABL1)* gene on chromosome 9, resulting in the constitutionally active tyrosine kinase, *BCR::ABL1*, and the Philadelphia chromosome t(9;22) (1). The downstream effect of the *BCR::ABL1* fusion is proliferation of myeloid cells. Following the advent of the tyrosine kinase inhibitor (TKI) imatinib, the mortality of patients with CML significantly improved, resulting in near-normal life expectancies (2). Although drugs targeting the kinase activity of *BCR::ABL1* are a highly effective treatment for CML, resistance can arise either through mutations in the *ABL1* kinase domain or through kinase-independent mechanisms. The etiology of kinase-independent resistance is incompletely described. Frequently, the presence of other myeloid malignancy-associated mutations is associated with kinase-independent resistance, although the mechanism of this is not completely understood (3).

Although mutations in the *BCR::ABL1* kinase domain are found in patients who have relapsed on TKI treatment, mutations in other genes have also been described (4–7). Recurrent mutations in Additional sex combs-like 1 *(ASXL1)*, Runt-related transcription factor 1 *(RUNX1)*, and Isocitrate dehydrogenase 1 (*IDH1*) have been identified in myeloid neoplasia, and mutations in these genes along with DNA (cytosine-5)-methyltransferase 3A (*DNMT3A)*, SET binding protein 1 (*SETBP1)*, and tumor protein 53 (*TP53)* are involved in the development of tyrosine kinase-independent resistance and progression to advanced disease stages in CML (8). These genes are also often associated with disease progression in CML (9). They have also been detected in CML clones that have persisted after optimal TKI response (10).

## Case report

In this case, the patient presented at 15 years of age with bone pain and splenomegaly. Routine laboratory values at the time showed a white blood cell (WBC) count of 28.8 × 10^3^/μl and a hemoglobin level of 10 g/dl. Fluorescence *in situ* hybridization (FISH) was positive for *BCR::ABL1*. She was started on imatinib at this time. A high level of the BCR-ABL p210 transcript was detected at 58% IS at diagnosis and then detected at 103% IS. There was no p190 detected at diagnosis or during disease course.

On diagnosis, she was started on Imatinib 400 mg daily and achieved complete hematologic response (CHR) at 6 months and complete cytogenetic response (CCR) at 9 months. Records of her treatment response during this time are incomplete, and it is unclear whether she ever achieved a major molecular response MMR. Subsequently, she developed side effects of Imatinib including rash and periorbital edema leading to discontinuation in 2013. She was started on Nilotinib and had myalgias leading to discontinuation after 3 months. The degree of response that she achieved on nilotinib is unknown. She was initiated on Dasatinib in 2016 but this did not lead to improvement of side effects as she developed a rash and asthenia. This was again discontinued after 6 months. In June 2018, she was re-trialed on Dasatinib, but she discontinued it due to intolerable side effects. The degree of response she achieved on Dasatinib is unknown. In July 2019, she presented with hyperleukocytosis and a WBC of 32 × 10^3^/μl and was treated with hydroxyurea. A bone marrow biopsy was performed, which was markedly hypocellular. She was restarted on Dasatinib 100 mg between July 2019 and March 2020. She was due for bone marrow transplant (BMT) but because of her intermittent adherence with TKIs, she was not a BMT candidate. While on Dasatinib, she developed persistent pancytopenia with platelet count as low as 11,000 despite dose reduction from 100 mg to 50 mg to 25 mg. She was given a course of steroids due to suspicion of immune thrombocytopenic purpura (ITP). She was transfusion dependent most of 2020. Dasatinib was discontinued due to pancytopenia. She was restarted on Nilotinib but again developed whole-body rash. Bone marrow biopsy in December 2020 showed CML with cellularity of 50% and hyper-lobulated megakaryocytes.

In March 2021, she transferred care to our center. She was initiated on Bosutinib 400 mg along with Eltrombopag in an effort to improve her transfusion-dependent thrombocytopenia. BCR–ABL1 PCR was 56% (IS) at the time. In May 2021, she underwent a bone marrow biopsy that was performed to evaluate her ongoing lack of molecular response and pancytopenia. This showed normal lymphocyte morphology and normal platelet morphology. Her granulocyte lineage showed a marked left shift; however, blasts were <1%, consistent with continued chronic phase. Next-generation sequencing (NGS) of the bone marrow aspirate showed the following mutations: ASXL1 p.Q976 alteration at 43% VAF, RUNX1 p.R201Q alteration at 27% VAF, and RUNX1 splice site alteration at 13% VAF. Given her lack of response to Bosutinib, she was switched to Ponatinib 30 mg. ABL sequencing was performed in April 2021 and May 2022 and both sequencing were negative for mutations.

**Figure 1:**
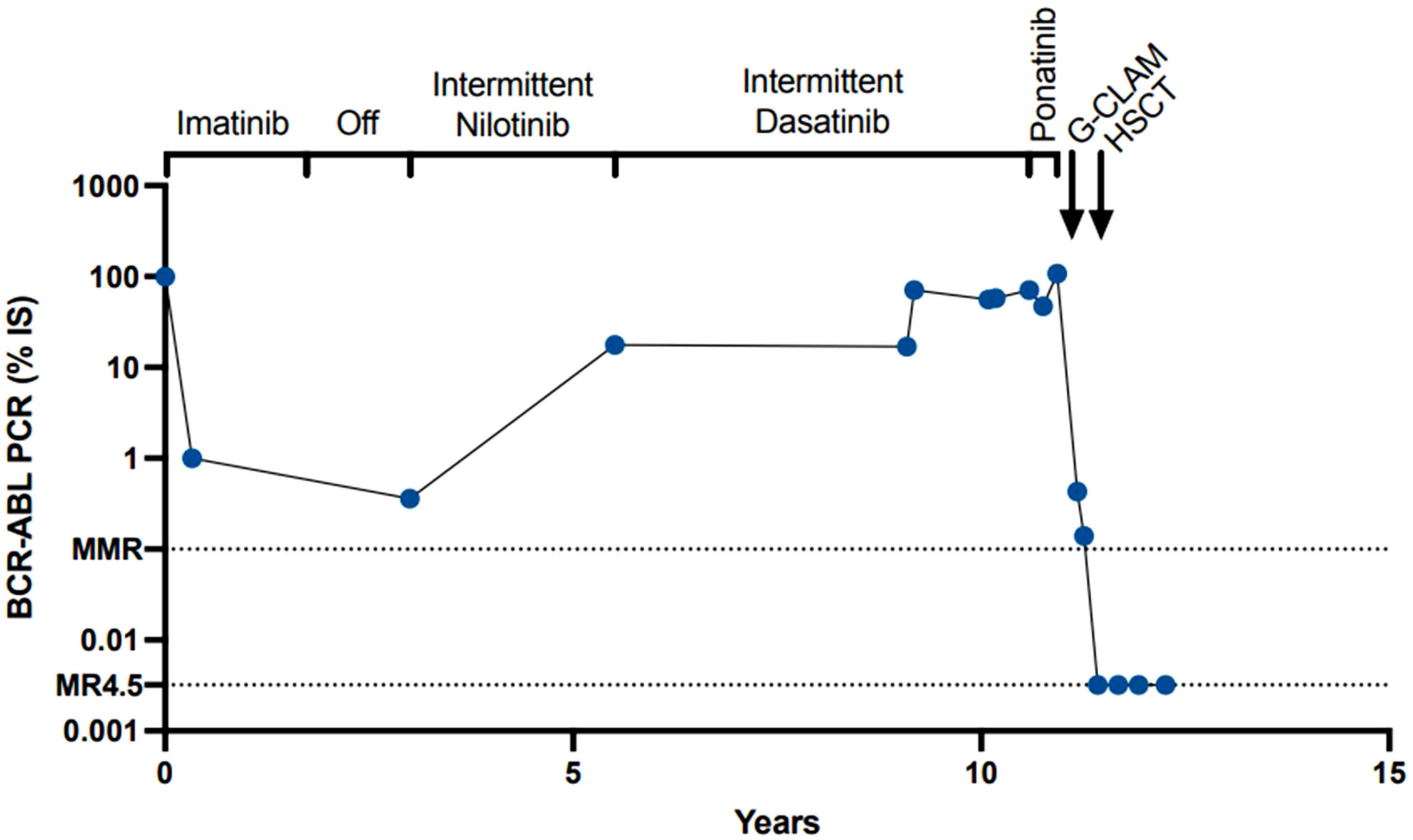
BCR-ABL1 levels through disease course.

Over the next year, she continued to have transfusion-dependent pancytopenia without evidence of molecular response to therapy (**Figure 1**). She was once again evaluated for BMT; however, this process was repeatedly delayed due to social issues. In the spring of 2022, she was undergoing the final stages of workup for bone marrow transplantation, and required multiple admission for abdominal pain, nausea, vomiting, menorrhagia, and abdominal bleeding. Further workup at the time revealed a hemorrhagic cyst in the right ovary and hemoperitoneum. During this time, she continued to exhibit transfusion-dependent pancytopenia without obvious changes in kinetics. Hemoglobin remained stable before and after cyst rupture between 7.7 and 8.2. She was started on oral contraceptive (progesterone norethindrone) and an intrauterine device (IUD) was placed. Despite the aforementioned interventions, her symptoms persisted. She underwent diagnostic laparoscopy, which showed dense adhesions and firmness of the distal sigmoid colon and proximal rectum. Endoscopy and colonoscopy showed a tortuous colon, and mucosal petechiae in the stomach and colon. A biopsy of the colonic mucosa was unremarkable. Her pelvic pain continued, and she was found to have a ruptured ovarian cyst. A repeat bone marrow biopsy showed a hypercellular (>90%) marrow with 58% monocytes, and 13% marrow blasts/blast equivalents, consistent with progression to a CMML-like accelerated phase. In addition to blasts from bone marrow, the patient also had clonal evolution and splenomegaly while on therapy. Patient had leukocytosis greater than 10 × 10^9^ (peak of 28.4). Basophils were not above 20%. NGS showed a new pathogenic *IDH1* p.R132H mutation (27% VAF) and the previously detected *RUNX1* splice site mutation (40% VAF), *RUNX1* p.R201Q (1% VAF), and *ASXL1* p.Q976 (39% VAF). During this hospitalization, she had symptoms of increased systemic inflammatory response including pleuritic chest pain and a large pericardial effusion with pericardial tamponade. Subsequent pericardiocentesis was performed with approximately 0.7 L of serous fluid initially removed and 4.22 L additionally drained. The etiology of pericardial tamponade was thought to be either pericarditis based on clinical presentation and electrocardiogram (ECG), or neoplastic, due to the abrupt onset of symptoms that coincided with progression to AP-CML. Pericardial fluid was sent for flow cytometric analysis and showed no evidence of lymphoma or acute leukemia with 37 monocytes seen on differential.

She underwent induction therapy with filgrastim, cladribine, cytarabine, and mitoxantrone (G-CLAM) for accelerated phase CML. Her bone marrow following G-CLAM treatment was hypocellular with no evidence of a myeloid neoplasm. NGS did not detect the previously identified *IDH1*, *RUNX1*, and *ASXL1* variants, and quantitative PCR for the *BCR::ABL1* transcript showed of 0.43% IS. As a bridge to BMT, she was started on ivosidenib, an IDH1 inhibitor.

She underwent stem cell transplantation from an unrelated donor. Currently, she is more than 100 days status post-transplant. Day +30 marrow studies showed a normocellular marrow with trilineage hematopoiesis and no increased blasts. Day +100 marrow studies showed hypocellular bone marrow with trilineage hematopoiesis, no increased blasts, and no definitive evidence of CML. Post-transplant, *BCR::ABL1* remains undetectable by qPCR. NGS on day 30 and day 100 did not detect the previously identified *IDH1*, *ASXL1*, and *RUNX1* variants. She achieved major molecular response (MMR) only after transplant. She did not achieve MMR while on TKIs.

## Discussion

**Figure 2:**
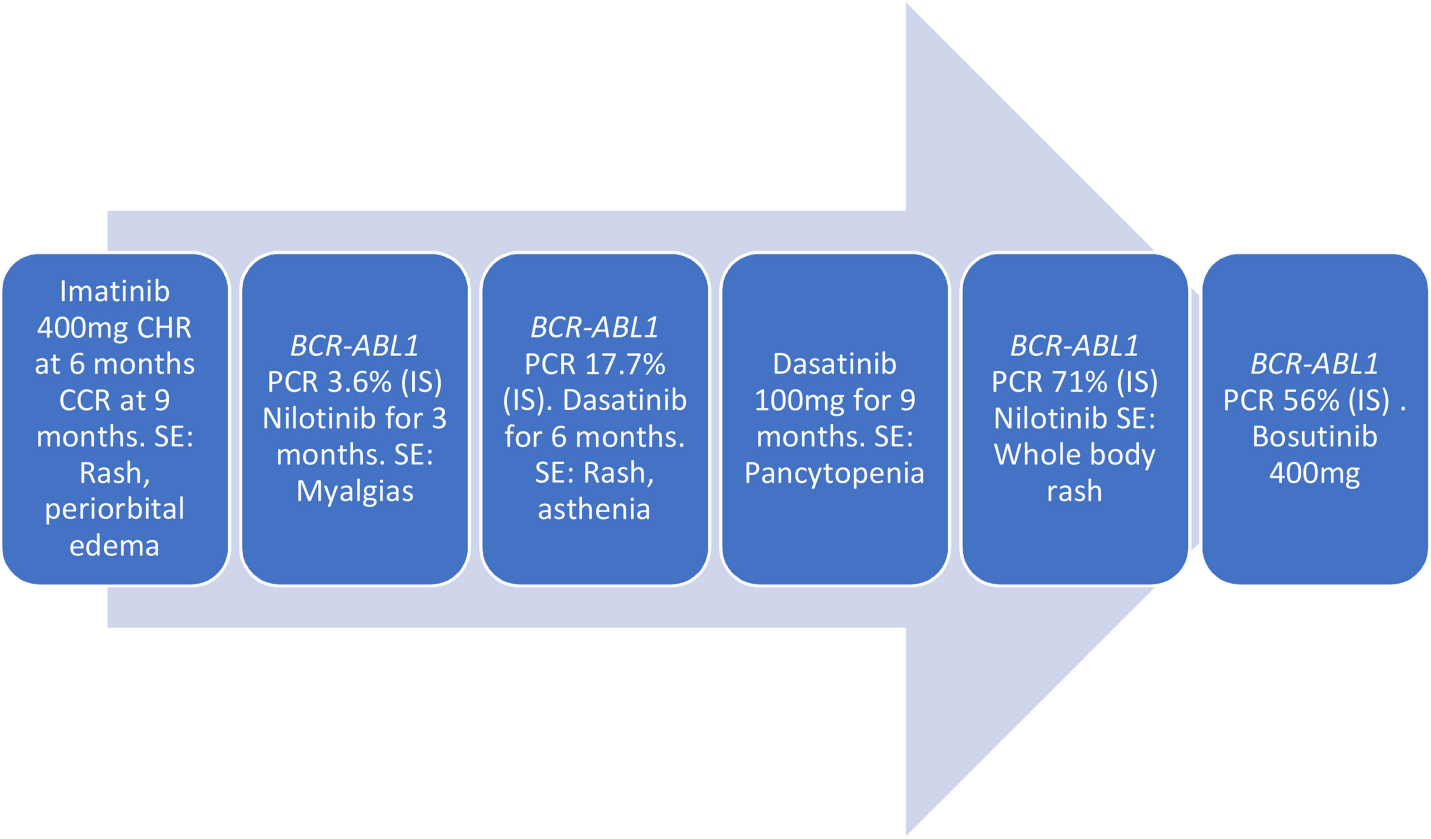
Tyrosine kinase inhibitors (TKIs) treatment course.

In this report, we present the case of a *BCR::ABL1* tyrosine kinase-independent resistance. It is well known that *BCR::ABL1* kinase domain mutations can be present in individuals who relapse on TKI treatment. However, there is evidence that there can be *BCR::ABL1* mutations independent of tyrosine kinase; TKI resistance can develop through pathways downstream of *BCR::ABL1* (10). In this case, although the patient was intermittently adherent to multiple TKIs due to side effects, while she was on the initial TKI, she did have clinical complete remission (CCR) with dropping *BCR::ABL1* to undetectable levels. It is unclear if the patient’s CML was aggressive at diagnosis at age 15 or if this was an evolution that occurred due to intermittent adherence to TKIs (**Figure 2**).

Subsequently, she achieved some level of disease control with the other TKIs but failed to achieve optimal response in the setting of intermittent adherence. Previous work has shown that *BCR::ABL1* kinase domain mutation resistance is also associated with the presence of mutations in the following epigenetic regulators: *ASXL1*, *DNMT3A*, *IDH1*, and *SETBP1* (4). Our patient had an *ASXL1* variant (p.Q976), which was present at diagnosis, and an *IDH1* variant, which was only present at progression. The IDH mutation is a mechanism of progression that resulted from natural disease progression in the setting of intermittent TKI adherence. In addition to variants in *ASXL1* and *IDH1*, a *RUNX1* variant was also present. Pathogenic *RUNX1* variants are known to contribute to progression of CML (11). At present, it is still unclear how these mutations drive kinase-independent resistance. Although one of the RUNX1 mutations was not present during the accelerated phase, the significance of this disappearance is unclear.

As mentioned in the case above, the patient also had nonspecific hematologic, gastrointestinal, and genitourinary findings, the etiology of which is still unknown. Extensive workup including surgical intervention was unrevealing. In addition, she also had pericarditis of unclear etiology. These symptoms all coincided with the appearance of a pathogenic *IDH1* mutation and progression to CMML-like AP-CML. CMML has been associated with a wide array of systemic inflammatory and auto-immune phenomena (12).

Although nonspecific systemic inflammatory and autoimmune diseases (SIADs) have not been described in CML, this phenomenon has been described in some other hematologic malignancies including MDS, CMML, and bone marrow failure syndromes (13). These can manifest as vasculitis, connective tissue disease, or inflammatory arthritis (14). In most cases, treatment with systemic steroids and/or hypomethylating agents leads to resolution of symptoms. The precise etiology of this systemic inflammation remains unclear. However, recent single-cell profiling work in CMML revealed that granulocyte macrophage progenitors (GMPs) with an inflammatory phenotype expand in CMML and are associated with disease progression (15). In the case of our patient, she developed an inflammatory pericarditis with effusion at the time of progression to accelerated phase. It is tempting to speculate that this was driven by CMML-like phenotype of her disease and the resulting inflammation. Consistent with this, her symptoms resolved after induction therapy and subsequent bone marrow transplantation.

**Figure 3:**
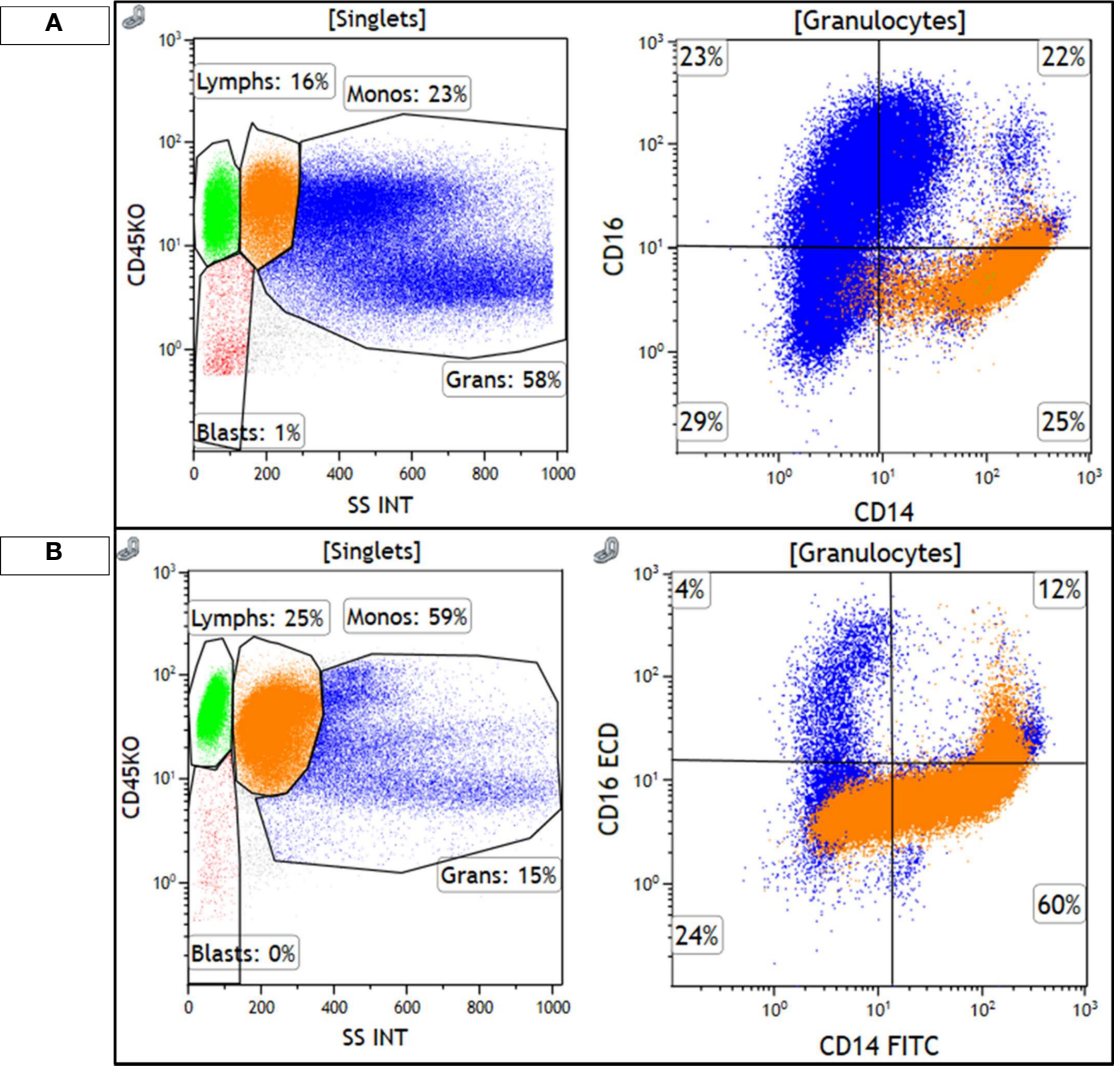
Monocytes are highlighted in orange and granulocytes are highlighted in blue. **(A)** shows the diagnostic, chronic phase marrow, with 23% monocytes that show a classical monocyte (M0) immunophenotype, CD14+/CD16−, with minimal loss of CD14 expression. Granulocytes are the majority of cellular events and show a spectrum of CD16 expression, consistent with the left-shifted myeloid series seen in CML. **(B)** shows the accelerated phase marrow, with 59% monocytes that show an abnormal phenotype, with some loss of CD14. Maturing granulocytes are decreased, and are mostly mature CD16+ neutrophils.

**Figure 4:**
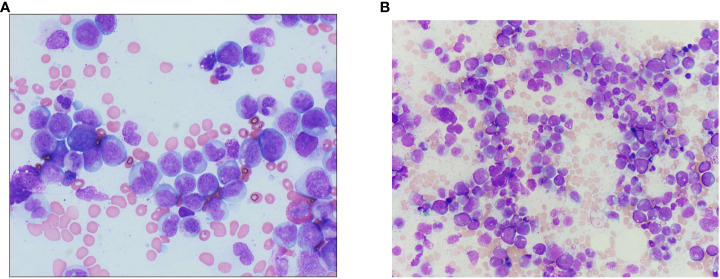
**(A)** Bone marrow aspirate from the accelerated phase marrow (Wright–Giemsa-stained preparation, 600× magnification), showing an increased number of monocytes and monocyte precursors, the latter of which show fine, dispersed chromatin and a lack of nuclear folding. **(B)** Wright–Giemsa-stained aspirate smears from diagnostic chronic phase marrow show left-shifted myeloid hyperplasia without increased blasts, with decreased erythroid precursors, consistent with the diagnosis of chronic myeloid leukemia.

We postulate that the development of a pathogenic variant in *IDH1* kinase-independent played a role in the disease progression described in this case. This variant has been previously described in tyrosine kinase-independent resistance and has been shown to predispose patients to progression of CML (4). Furthermore, the development of pathogenic variant in *IDH1* that led to disease progression also caused CMML-related inflammatory response as seen in her AP clone showing CMML-like morphology and immunophenotype (**Figure 3**, **4**). CMML has been associated with a wide array of systemic inflammatory and auto-immune phenomena (12). Similar to the syndrome of non-specific SIADs present in CMML, this case also showed a constellation of systemic inflammatory processes during progression to AP-CML, including a serous pericardial effusion. It seems probable that the systemic findings in this case could be secondary to CMML-driven systemic inflammation.

## Conclusion

This case highlights tyrosine kinase-independent CML with a typical mutational profile including pathogenic variants in *ASXL1* and *RUNX1*, with acquisition of an *IDH1* mutation at the development of a unique CMML-like AP. It highlights the need for a better understanding of how somatic mutations influence response to TKIs, enabling the development of new treatment approaches for kinase-independent resistance. To our knowledge, nonspecific SIADs have not been described in CML.

The limitations of our work include the fact that this is a case report with a sample size of one; hence, there is a lack of ability to generalize. In spite of the singular sample size, our case represents a novel presentation of kinase-independent resistance in CML.

## Data availability statement

The original contributions presented in the study are included in the article/supplementary material. Further inquiries can be directed to the corresponding author.

## Ethics statement

Written informed consent was obtained from the individual(s) for the publication of any potentially identifiable images or data included in this article.

## Author contributions

EO wrote the manuscript. LS reviewed manuscript and provided figures. PR reviewed manuscript and provided figures. TB wrote and reviewed manuscript. All authors contributed to the article and approved the submitted version.
